# TTK regulates proliferation and apoptosis of gastric cancer cells through the Akt‐mTOR pathway

**DOI:** 10.1002/2211-5463.12909

**Published:** 2020-07-01

**Authors:** Hongxia Huang, Yadong Yang, Wenyuan Zhang, Xinzhu Liu, Geng Yang

**Affiliations:** ^1^ School of Medicine Zhejiang University City College Hangzhou China; ^2^ Institute of Bioengineering Hangzhou Medical College Hangzhou China

**Keywords:** Akt‐mTOR pathway, apoptosis, gastric cancer, TTK

## Abstract

TTK (also known as Mps1) is the core component of the spindle assembly checkpoint, which ensures proper distribution of chromosomes to daughter cells to maintain genome integrity and to balance growth and division. However, the function of TTK in tumorigenesis has not been extensively studied, especially in relation to the development of gastric cancer. In this study, survival and tumor recurrence data related to TTK expression level in gastric cancer patients were collected and analyzed. We observed that TTK expression was negatively correlated with survival and tumor recurrence *in vivo*. TTK was also upregulated in gastric cancer cells and was observed to be essential for the proliferation and survival of gastric cancer cells. Knockdown of TTK inhibited proliferation and increased apoptosis. Furthermore, we report that TTK regulates the proliferation and apoptosis of tumor cells through the Akt‐mTOR pathway. Knockdown of TTK inhibited activation of Akt‐mTOR signaling. In summary, our data indicate that TTK is involved in the regulation of gastric cancer proliferation and apoptosis.

## Graphical abstract

We report here that high TTK expression is correlated with lower survival rate and higher tumor recurrence in gastric cancer patients. Knockdown or inhibition of TTK in gastric cancer cells resulted in Akt‐mTOR dysregulation and enhanced apoptosis of cancer cells. Our data suggest that TTK is involved in the regulation of gastric cancer proliferation and apoptosis.

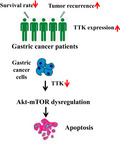

**Abbreviations**
ANOVA: analyzed by one‐way analysis of variance
HMPS1/TTK: the human monopolar spindle 1
mTOR: the mammalian target of rapamycin
PVDF: polyvinylidene difluoride
SAC: the spindle assembly checkpoint
SD: standard deviation

Gastric cancer is one of the most common tumors in the digestive tract. The incidence rate of gastric cancer is fourth among malignant tumors, and it has a high mortality rate [1]. Studies have shown that the carcinogenic process is very complex, which results from continuous accumulation of genetic mutations. Currently, the specific pathogenesis of gastric cancer is unclear [2, 3]. Conventional chemotherapy and biotherapy are not ideal. Therefore, studies on the pathogenesis of gastric cancer and clinical treatment have become the focus of research [4, 5, 6].

Protein kinases are catalytically active enzymes that transmit functional signals by regulating important cellular processes. Dysfunction of kinases in cells often leads to the development of cancer and is associated with tumor progression and clinical outcomes [7]. Therefore, studies on kinases have facilitated screening of new inhibitors for the treatment of cancer. For example, sorafenib is a multitargeted kinase inhibitor proven to an effective inhibitor in both advanced liver cancer and kidney cancer [8, 9]. Apatinib, a small molecule tyrosine kinase inhibitor targeting vascular endothelial growth factor receptor‐2, has been recommended as third‐line treatment for metastatic gastric cancer patients [10].

The human monopolar spindle 1 (HMPS1/TTK) gene is located on chromosome 6q13‐q21 and encodes a serine/threonine and tyrosine protein kinase. TTK is critical for the regulation of mitotic checkpoints and chromosome attachment. Elevated levels of TTK cause centrosome enlargement and chromosomal instability, leading to tumorigenesis [11, 12, 13]. Potential diagnostic value of TTK has been demonstrated for undifferentiated thyroid, triple‐negative breast, and lung cancers [14, 15, 16]. It has been reported that specific inhibitors of TTK, AZ3146 and MPI‐0479605, effectively have been described to inhibit proliferation of hct‐116 colon cancer cells, indicating that TTK is involved in tumor formation [17, 18, 19]. However, the relationship between TTK and the formation of gastric cancer has not been reported in the literature, and its role and molecular mechanism still need further study.

In this study, we found that the expression of TTK was positively correlated with the mortality rate of patients with gastric cancer, and high expression of TTK was conducive for tumor recurrence. We also found that overexpression of TTK promoted cell proliferation, and inhibition or knockdown of TTK promoted apoptosis, indicating that TTK plays a role as an oncogene during tumor formation. Further experiments revealed that TTK regulated cell proliferation and apoptosis by participating in the Akt‐mTOR signaling pathway.

## Material and methods

### Cell culture and transfection

Immortalized gastric cell line GES‐1 and cancer cell lines HGC‐27 and MGC‐803 were cultured and maintained at 37 °C with 5% CO_2_ in 1640 medium (HyClone, Logan, UT, USA) containing 10% FBS (Gibco, Brisbane, Australia). Cells were cotransfected with plasmids expressing TTK or siRNA according to the manufacture's instruction. The siRNAs used for TTK knockdown were TTK‐siRNA‐1 (5′‐AGAUCUUCGAGACGUGUU AAA‐3′) and TTK‐siRNA‐1 (5′‐AGAUUGAAUUUCCCGAGAUUU‐3′). Briefly, when cells reached about 70% confluence in a 6‐well plate, 2 µg for each indicated plasmid and 6 µL Lipo2000 Reagent were diluted in 100 µL 1640 medium, respectively. The diluted plasmids and Lipo2000 Reagent were then mixed gently and incubated for 30 min at room temperature. The mixture was added to the cells and incubated in a 37 °C incubator for 6 h before the medium was replaced by fresh 1640 medium. Cells were finally subjected to further research 48 h after transfection. For siRNA transfection, cells were cultured to about 50% confluence in a 6‐well plate and the indicated siRNA was transfected by Lipo2000 Reagent at the final concentration of 100 nm. The cells were incubated with siRNA for about 48 h before the determination of TTK expression by western blot. MGC‐803 or HGC‐27 was used for the transfection of siRNAs to knock down TTK expression.

### Plasmid construction

Total RNA was extracted from HeLa cells using an RNA Isolation Kit (Ambion, Austin, TX, USA). The reverse transcription reaction was conducted using PrimeScript RT Reagent Kit (Takara, Kyoto, Japan), and the cDNA was then used as the template for the amplification of TTK gene. The ORF of TTK was amplified by the following sequences: pcDNA‐Flag‐TTK‐F:GGAggatccATGGATTACAAGGACG ACGATGACAAGATGGAATCCGAGGATTTAAGTG.PcDNA‐Flag‐TTK‐R:GGTctcgagTCAAAAAGTCTTGGAGGATGAA. The fragment was then digested and cloned into the plasmid (Invitrogen, Carlsbad, CA, USA). GES‐1 was used for the transfection of Flag‐TTK.

### Quantitative real‐time PCR for the detection of gene expression

Real‐time PCR was used to quantify gene expression level in cells. RNA was extracted from cells using RNA Isolation kit (Ambion) and reversely transcribed into cDNA (Takara). Gene expression was then determined using gene‐specific primers (5′‐TAGCCCAGATTGTGATGTGAAG‐3′ and 5′‐CACTTGGTTTAGATCCAGGCAC‐3′); the 25 μL PCR solution contained 12.5 μL of 2 × Premix Ex Taq (TaKaRa), 0.5 μL of 10 μm forward primer, 0.5 μL of 10 μm reverse primer, and 1 μL DNA template. The stages of PCR program were set as 95 °C for 5 min, followed by the amplification stage consisting of 40 cycles of 95 °C for 10 s and 60 °C for 30 s.

### Western blot analysis

12% SDS/PAGE was used to separate proteins, which were then transferred onto a polyvinylidene difluoride (PVDF) membrane (Millipore, St. Louis, MO, USA). The membrane was blocked by 5% nonfat milk in phosphate‐buffered solution (NaCl 137 mm, KCl 2.7 mm, Na_2_HPO_4_ 10 mm, KH_2_PO_4_ 2 mm, pH 7.4) for 2 h at room temperature. The blot was incubated with a primary antibody (Table S1) at 4 °C overnight in a flat shaker. After washed by PBS, the blot was incubated with the fluorescence‐labeled anti‐mouse/anti‐rabbit IgG (Cell Signaling Technology, Beverly, MA, USA) for 2 h at 4 °C. The blot was detected by Odyssey (Licor, Lincoln, NE, USA) for signals.

### Cell proliferation assay

MTS was used to measure cell proliferation according to the instruction (Promega, Madison, WI, USA). The cells were cultured in 96‐well plate. After the indicated treatments, the supernatant was replaced by fresh medium. Twenty microlitre of CellTiter 96 AQueous One Solution Reagent was added into each well of the 96‐well assay plate containing the samples in 100 µL of culture medium. The plate was incubated at 37 °C for 1 h in a humidified, 5% CO_2_ atmosphere. The absorbance was recorded at 490 nm using a 96‐well plate reader.

### Cell viability assay

CellTiter‐Glo® Luminescent Cell Viability Assay was used to measure cell viability according to the instruction (Promega). Cells were cultured in 96‐well plated with the indicated treatment. The plate and its content were equilibrated at room temperature for approximately 30 min. One volume of CellTiter‐Glo® Reagent equal to the volume of cell culture medium was added and mixed for 2 min on an orbital shaker to induce cell lysis. The absorbance was recorded at 490 nm using a 96‐well plate reader.

### Annexin V for the detection of gastric cancer cell death

Cell death rate was detected by Annexin V (Molecular Probes, Carlsbad, CA, USA) according to the manufacturer's protocol. Gastric cells were harvested after the indicated treatments and washed with cold phosphate‐buffered saline (NaCl 137 mm, KCl 2.7 mm, Na_2_HPO_4_ 10 mm, KH_2_PO_4_ 2 mm, pH 7.4). The cells were then resuspended in 1× annexin‐binding buffer (50 mm HEPES, 700 mm NaCl, 12.5 mm CaCl_2_, pH 7.4) at ~ 1 × 10^6^ cells per mL, followed by the addition of 5 μL of Alexa Fluor 488 annexin V and 1 μL of 100 μg·mL^−1^ PI (propidium iodide). The sample was incubated at room temperature for 15 min in the dark. Subsequently, 400 μL of 1× annexin‐binding buffer was added to the sample and measured by flow cytometry at 530 and 575 nm.

### TdT‐mediated dUTP nick end labeling (TUNEL) assay

TUNEL system (Promega) was conducted according to the manufacturer's instruction with some minor modifications. Briefly, the medium of cells was removed after the indicated treatment. The cells were fixed with 1% methanol‐free paraformaldehyde for 10 min at 4 °C. Subsequently, the cells were rinsed twice with PBS and permeabilized with 0.1% Triton X‐100 for 5 min. After rinsing slides with PBS twice at room temperature, the cells were covered with 100 μL of equilibration buffer at room temperature for 10 min. The cells were incubated with rTdT mix containing green fluorescein‐12‐dUTP for 60 min at 37 °C and then counterstained with PI. The reactions were terminated by immersing the slide in 2× SSC for 15 min. The slide was air‐dried and mounted with antifade solution (Invitrogen) to assess fluorescence by microscopy.

### Caspase 3/7 activity detection

The Caspase‐Glo 3/7 assay (Promega) was used to measure caspase‐3 and caspase‐7 activities according to the instruction. Briefly, the Caspase‐Glo 3/7 buffer was transferred to the amber bottle containing Caspase‐Glo 3/7 substrate to form the Caspase‐Glo 3/7 reagent. Cells with the indicated treatments were mixed with equal volume of reagent. The mixture was gently mixed and incubated at room temperature on a plate shaker for 2 h and then subjected to measurement of the luminescence by a plate‐reading luminometer.

### Statistical analysis

The data from three independent experiments were analyzed by one‐way analysis of variance (ANOVA) to calculate the mean and standard deviation (SD) of the triplicate assays.

## Results

### Relationship between TTK gene mutation and survival rate and recurrence of gastric cancers

**Fig. 1:**
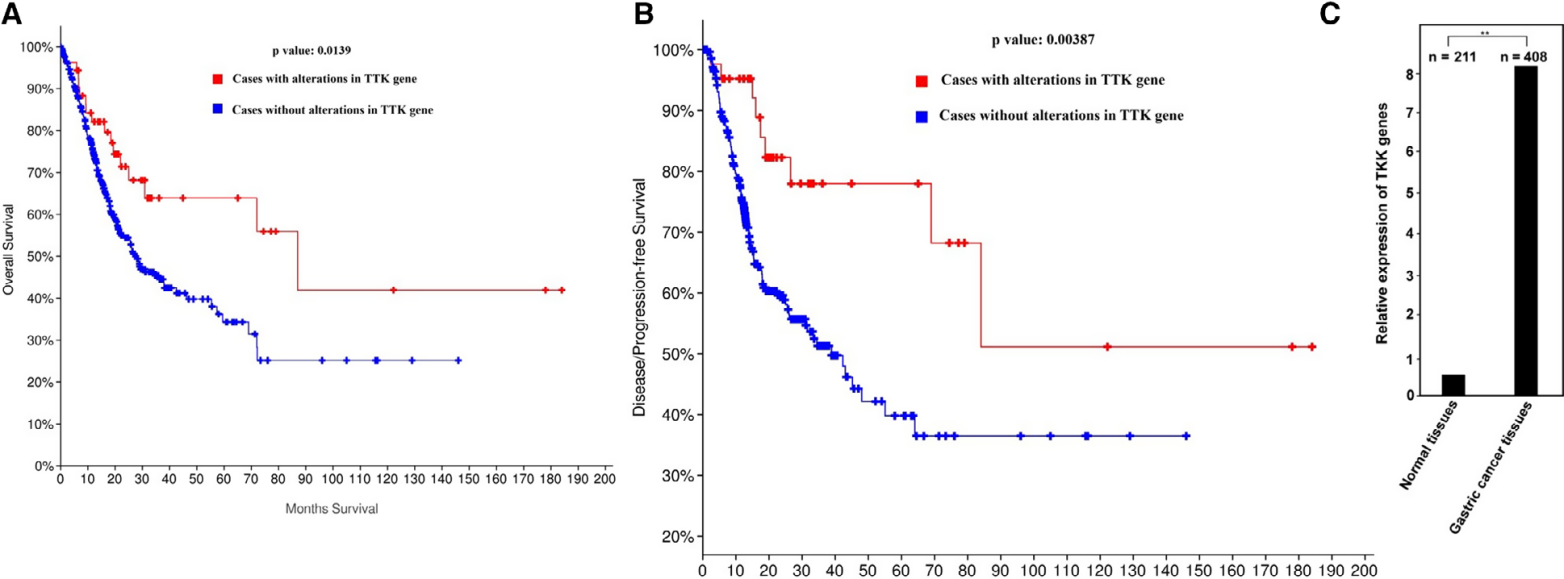
Survival and disease‐/progression‐free of the patients with or without TTK alteration. (A) Survival of the patients with or without TTK alteration. *P*‐value = 0.0139. (B) Disease‐/progression‐free survival of patients with or without TTK alteration. *P*‐value = 0.00387. All patient data were collected from TCGA database. (C). TTK expression profile in gastric cancer samples and paired normal tissues. Data represent means ± SD of triplicate assays and were analyzed by unpaired *t*‐test. ** *P* < 0.01.

First, to analyze whether TTK gene mutation is associated with the survival rate and recurrence of cancer patients, we searched for data on gastric cancer patients in the TCGA database. The database search revealed 92 gastric cancer patients with TTK mutations with an average survival period of 87 months. However, in 397 gastric cancer patients who did not have a mutated TTK gene, their mean survival was only 27.33 months (Table 1). By analyzing the survival rate of gastric cancer patients, we also found that the survival rate of cancer patients with TTK mutations was significantly higher than that of cancer patients without TTK gene mutations (Fig. 1A). This result preliminarily indicated that the TTK gene may function as an oncogene. Next, we investigated the relationship between TTK mutation gene and gastric cancer recurrence. We searched the data on cancer patients who underwent chemotherapy or surgical resection and analyzed the relationship between tumor recurrence and survival rate. As shown in Table 2, in the TTK gene mutation group, the tumor recurrence rate was about 18%, and the average survival period was 98 months. In the unmutated TTK group, the tumor recurrence rate was about 37%, and the average survival period was only 38.9 months. Figure 1B also shows that cancer patients with TTK gene mutations had a longer tumor recurrence time and higher survival rate than those without TTK mutation, indicating that TTK participates in the tumor recurrence process. In order to further analyze the gene expression profile across gastric cancer samples and normal tissues, we searched the corresponding data from GEPIA (http://gepia.cancer‐pku.cn/). By analyzing TTK expression in 408 gastric cancer tissues and 211 normal tissues, we found that TTK was extremely upregulated in gastric cancers by about 16‐fold (Fig. 1C).

**Table 1:** Statistics of patients with or without TTK gene mutations in tumors. *P*‐value of these two groups is 0.0139.

	Number of cases, total	Number of cases, deceased	Median month survival
Cases with alterations in TTK gene	120	34	87
Cases without alterations in TTK gene	397	168	27.33

**Table 2:** Disease‐free survival of patient with or without TTK gene mutations in tumors. *P*‐value of these two groups is 0.00387.

	Number of cases, total	Number of case, relapsed/progressed	Median month survival
Cases with alterations in TTK gene	98	18	98
Cases without alterations in TTK gene	305	112	38.9

Together, these results indicate that TTK may function as oncogene and be of great importance in the tumorigenesis of gastric cancers.

### Effects of overexpression of TTK on cell proliferation and viability

**Fig. 2:**
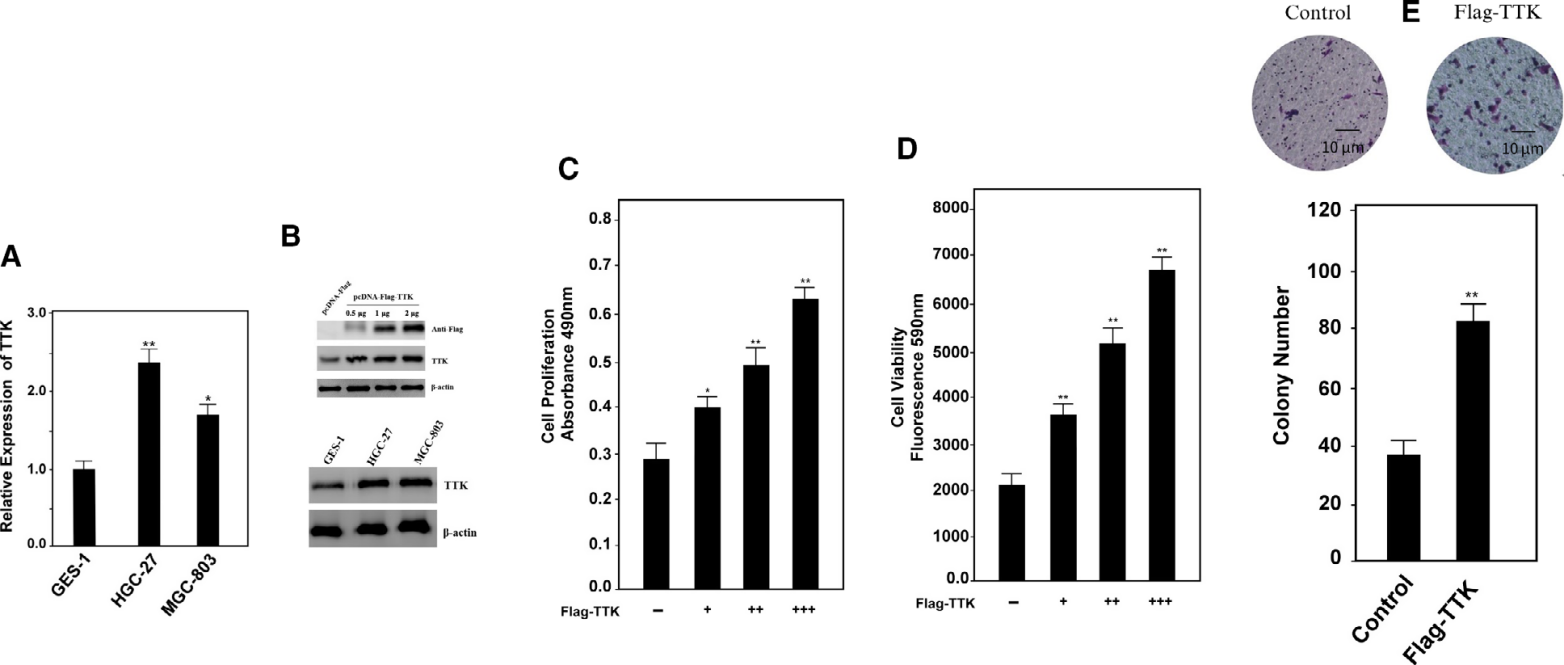
Ectopically expressed TTK enhances the growth and viability of gastric cancer cells. (A) mRNA and protein levels of TTK were detected by quantitative real‐time PCR (left) and western blotting (right), respectively, in immortalized gastric cells and gastric cancer cells. β‐actin was used as a control. Data represent means ± SD of triplicate assays and were analyzed by unpaired *t*‐test. *P < 0.05, **P < 0.01 (B) TTK expression in cells transfected with increasing amounts of plasmids, 1 µg(+), 2 µg(++), and 4 µg(+++), was detected by western blotting. β‐actin was used as a control. (C) Cell proliferation was determined by MTS assay after transfection of increasing amounts of the TTK expression plasmid. Data represent means ± SD of triplicate assays and were analyzed by unpaired *t*‐test. *P < 0.05, **P < 0.01. (D) Cell viability was determined after transfection of increasing amount of TTK expression plasmid. **P < 0.01. Data represent means ± SD of triplicate assays and were analyzed by unpaired *t*‐test. (E) Colony assay was performed, and the colony number was calculated after cells were transfected with or without Flag‐TTK. In all of the panels, the plotted data points refer to the mean ± SD of triplicate assays. Data represent means ± SD of triplicate assays and were analyzed by one‐way ANOVA with Dunnett's multiple comparisons test. The scale bar indicated 10 μm in length.

To further investigate the specific function of TTK in cells, we examined the expression of TTK in various gastric cancer cells. Compared with immortalized gastric epithelial cell line GES‐1, we found that TTK was significantly overexpressed in gastric cancer cell lines HGC‐27 and MGC‐803 (Fig. 2A). We next inserted TTK cDNA in a plasmid vector with a Flag tag. After transfection of the plasmid into the cells, we performed western blotting on the TTK protein, which was found to be efficiently expressed in the cells. In addition, the TTK protein was increased as more plasmids were transfected (Fig. 2B). Next, we analyzed cell growth and viability upon overexpression of TTK. MTS assay showed that, as the expression level of TTK protein was increased, the proliferation rate of cells was also increased gradually (Fig. 2C). Further analysis revealed that TTK overexpression enhanced cell viability (Fig. 2D). In order to determine the impact of TTK expression on long‐term cell survival, colony assay was performed. The results showed that more colonies were formed on agarose plate after TTK was overexpressed in cells (Fig. 2E). Collectively, these results indicated that overexpression of TTK was beneficial for cell proliferation and tumor growth.

### Cell proliferation and apoptosis after knockdown of TTK

**Fig. 3:**
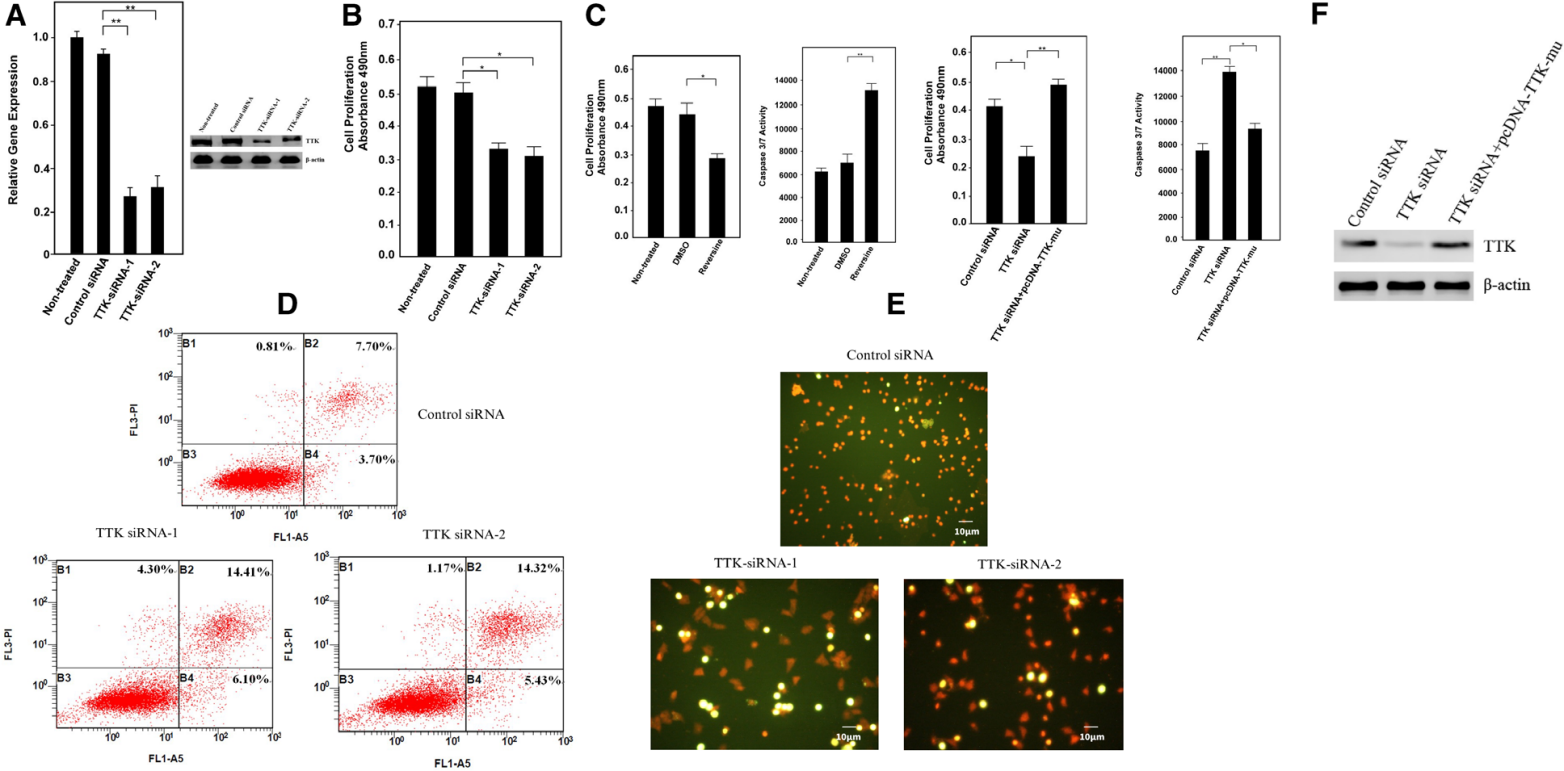
Depletion of TTK inhibits cell growth and induces apoptosis of gastric cancer cells. (A) TTK expression was evaluated by real‐time PCR (left) and western blotting (right) after cells were transfected with two different siRNAs. (B) Cell proliferation was determined by MTS assay after knockdown of TTK by siRNA transfection. *P < 0.05. (C). Cell proliferation or apoptosis was determined by MTS assay or caspase‐3/7 activity after treated with or without TTK inhibitor reversine. *P < 0.05. (D) Cells with or without knockdown of TTK were collected and stained with Annexin V and PI. The cells were analyzed by flow cytometry. (E) Effect of TTK1 reduction on the gastric cell death examined by TUNEL staining. Cells with or without knockdown of TTK were collected for the TUNEL assay. Samples were examined by fluorescent microscopy. The scale bar indicated 10 μm in length. (F) Cell proliferation was determined by MTS assay after transfection of siRNA with or without pcDNA‐TTK‐mu. *P < 0.05, **P < 0.01. (G) Apoptosis was determined by caspase‐3/7 activity after transfection of siRNA with or without pcDNA‐TTK‐mu. *P < 0.05, **P < 0.01. (H). The expression of TTK with the indicated treatments was determined by western blot. β‐actin was used as a control. In all of the panels, the plotted data points refer to the mean ± SD of triplicate assays. In all panels, data represent means ± SD of triplicate assays and were analyzed by unpaired *t*‐test.

We designed two siRNAs targeting TTK. After siRNA transfection into gastric cancer cells, the mRNA and protein expression of TTK were detected by real‐time PCR and western blotting, respectively. Both siRNAs effectively knocked down TTK in cells (Fig. 3A). Next, we examined the proliferation of the cells by MTS assays. When TTK was knocked down, the proliferation rate of the cells was significantly inhibited (Fig. 3B). The proliferation rate was also decreased while apoptosis increased upon the inhibition of TTK by reversine, an inhibitor which can specifically inhibit TTK activity (Fig. 3C). To investigate whether knockdown of TTK induced apoptosis, we first examined early and late apoptosis of cells by Annexin V and TUNEL staining, respectively. The results showed that, when TTK was knocked down, the number of early apoptosis of the cells was increased significantly (Fig. 3D). Similarly, TUNEL staining also showed cells were undergoing apoptosis upon knockdown of TTK (Fig. 3E). To demonstrate that TTK, but not other proteins caused the above phenomena, we silent‐mutated the TTK siRNA‐binding site and cotransfected it with siRNA into gastric cancer cells. By detecting cell proliferation and caspase‐3/7 activities, we found that expression of TTK gene effectively restored the growth rate of gastric cancer cells and inhibited apoptosis (Figs. 3F,G). The expression of TTK with the indicated treatments was determined by western blotting (Fig. 3H). These results indicated that the TTK gene was involved in cell proliferation. Moreover, when TTK protein was decreased, cells underwent apoptosis, indicating that TTK is an oncogene, which is involved in the regulation of gastric cancer tumorigenesis.

### Pathways of TTK regulating cell proliferation and apoptosis

**Fig. 4:**
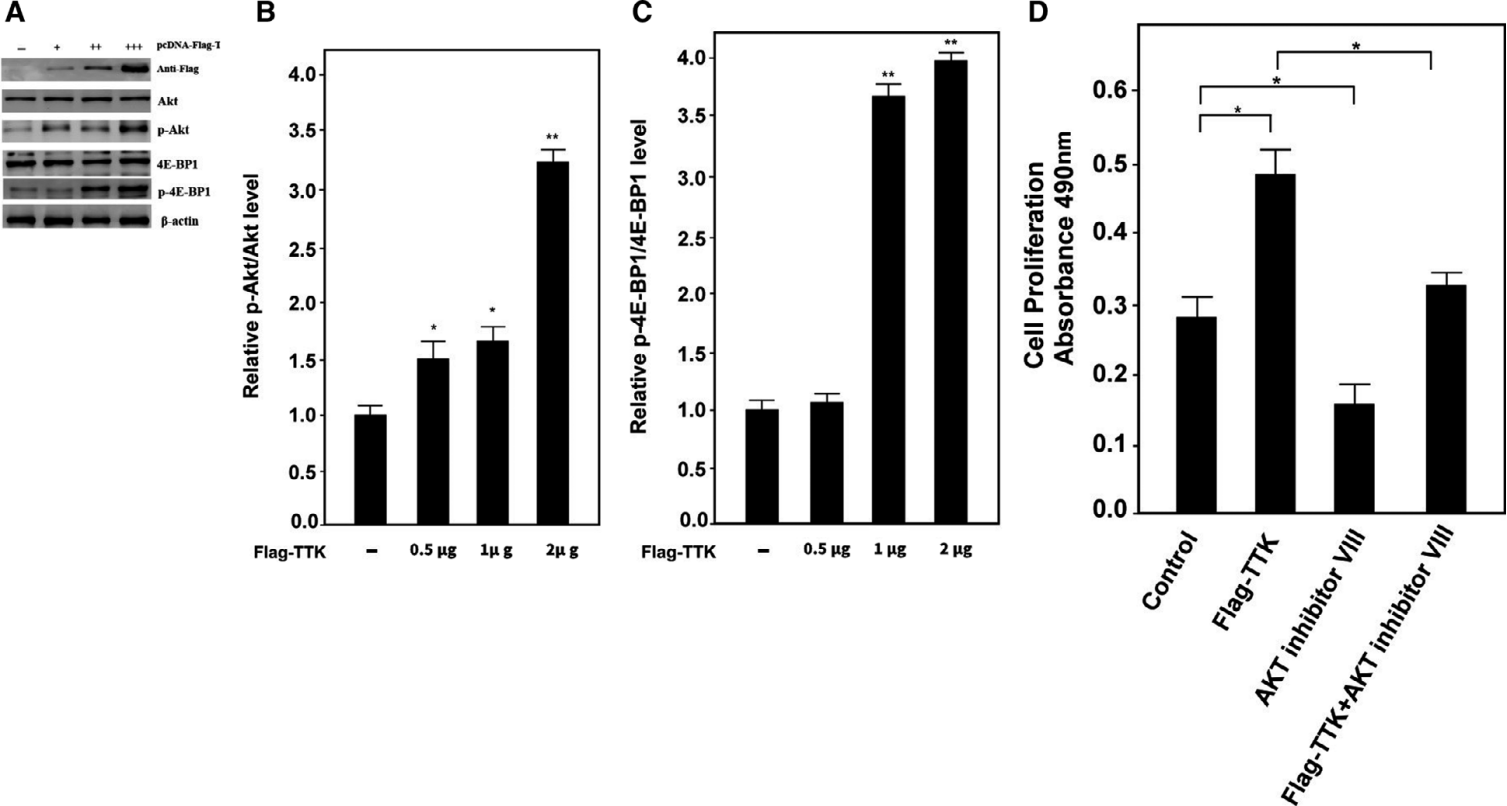
TTK participates in the Akt‐mTOR pathway to regulate cell growth and apoptosis (A) Akt, p‐Akt, 4E‐BP1, and p‐4E‐BP1 levels were determined by western blotting after cells were transfected with increasing amounts of TTK. β‐actin expression plasmid. β‐actin was used as a control. (B) Relative levels of p‐Akt/Akt were determined by the software of the Gel Imaging System (Bio‐Rad, Hercules, CA, USA). (C) Relative levels of p‐4E‐BP1/4E‐BP1 were determined by the software of the Gel Imaging System (Bio‐Rad). (D) Cell proliferation was determined by MTS assay after the indicated treatments. In all of the panels, the plotted data points refer to the mean ± SD of triplicate assays. In all of the panels, the plotted data points refer to the mean ± SD of triplicate assays. *P < 0.05, **P < 0.01.

The mammalian target of rapamycin (mTOR) is a serine/threonine kinase. The mTOR signaling pathway promotes cell proliferation and participates in many key physiological functions such as apoptosis and autophagy. It also plays a major role in many diseases. AKT kinase is one of the key proteins that regulates the mTOR signaling pathway [20]. Therefore, we investigate whether TTK protein regulates proliferation and apoptosis of tumor cells by modulating the Akt‐mTOR signaling pathway. First, we transfected increasing concentrations of the pcDNA‐Flag‐TTK plasmid into cells and then performed western blotting for the detection of Akt protein phosphorylation. The phosphorylation level of Akt protein was increased with the increased TTK protein expression (Figs. 4A,B). Next, we examined whether Akt directly activates mTOR pathway signaling by a downstream protein of the mTOR, 4E‐BP1. The results showed that the phosphorylation level of 4E‐BP1 protein was gradually increased with the increase of TTK expression (Figs. 4A,C). In order to further determine the connection between TTK and AKT, TTK was overexpressed with or without the incubation of AKT inhibitor VIII. The cell viability was then examined 48 h after the incubation, and the result showed that inhibition of AKT could block the acceleration of cell growth by TTK overexpression (Fig. 4D).

The above results indicated that TTK activated the Akt pathway that in turn regulated cell proliferation and apoptosis.

## Discussion

The spindle assembly checkpoint (SAC; also known as the mitotic checkpoint) is a signal cascade consisting of proteins such as TTK, polo, aurora, bub, bubr, and mad. Its function is to detect incorrectly located chromosomes, to produce the correct bipolar connection of the spindle, and reduce chromosome mismatches before the start of division [21]. TTK as a core component of SAC is a key monitoring mechanism that ensures healthy cell proliferation and precise division [22]. In addition to regulation of mitosis, TTK also plays a role in other processes such as centrosome replication, DNA damage response, and organ development [23]. Therefore, abnormal expression of TTK necessarily affects normal physiological functions, which may lead to serious chromosomal mismatch errors, eventually leading to chromosomal instability, aneuploid formation, and even cell cancer. By northern blot analysis, the expression levels of TTK genes in normal organs were very low except for testis and placenta. However, the level of TTK is high in many types of human malignancies, including glioblastoma, thyroid cancer, and breast cancer. These experimental results indicate that TTK is a typical proto‐oncogene. However, in addition to the clear study in cell mitosis, TTK protein has not been extensively studied in the specific mechanism of tumorigenesis, especially in the development of gastric cancer.

Our study found that TTK is associated with poor survival and poor prognosis in patients with gastric cancer and is expected to be a potential target for the treatment of gastric cancer. The expression of TTK kinase in gastric cancer is significantly higher than that of normal gastric immortalized cells, and its function is crucial for the proliferation and survival of gastric cancer cells. Knockdown of TTK results in decreased cell proliferation and increased rates of apoptosis and necrosis. Our results indicate that TTK regulates the proliferation and apoptosis of tumor cells through Akt‐mTOR signaling pathway. Knockdown of TTK protein inhibits the activation of Akt‐mTOR signaling pathway and reveals the mechanism of TTK involvement in tumor formation.

TTK could be a new marker for the prognosis and potential therapeutic targets of gastric cancer. So far, studies on tumors in combination with TTK inhibitors and chemotherapy or radiotherapy have yielded many encouraging results in the clinic. Therefore, more and more researchers are paying attention to this field, which may be a promising therapeutic target for various cancers. In addition, a new approach to the differential diagnosis of TTK between normal tissue and malignant tissue was observed. More specific studies are needed to validate this possibility. There is increasing evidence that overexpressed TTK is associated with shorter recurrence time and survival time. This suggests that TTK may also be an independent biomarker of prognosis. In conclusion, TTK may have potential as a therapeutic target and molecular biomarker.

## Conflict of interest

The authors declare no conflict of interest.

## Author contributions

YY, WZ, and XL carried out laboratory and data analysis. GY conceived this study and drafting the manuscript. HH supervised laboratory work and corrected the manuscript.

## Supporting information


**Table S1**. Antibodies used in this study.

## References

[1] Patru CL , Surlin V , Georgescu I and Patru E (2013) Current issues in gastric cancer epidemiology. Rev Med Chir Soc Med Nat Iasi 117, 199–204. 24505915

[2] Malcolm GS , Georgina LH , Eiichi T and Emad ME (2006) Cellular and molecular aspects of gastric cancer. World J Gastroenterol 12, 2979–2990. 16718776 10.3748/wjg.v12.i19.2979 PMC4124370

[3] Milne AN , Carneiro F , O’Morain C and Offerhaus GJA (2009) Nature meets nurture: molecular genetics of gastric cancer. Hum Genet 126, 615–628. 19657673 10.1007/s00439-009-0722-x PMC2771140

[4] Khan FA and Shukla AN (2006) Pathogenesis and treatment of gastric carcinoma: “an up‐date with brief review”. J Cancer Res Ther 2, 196–199. 17998703 10.4103/0973-1482.29830

[5] Lastraioli E , Romoli MR and Arcangeli A (2012) Immunohistochemical biomarkers in gastric cancer research and management. Int J Surg Oncol 24, 868645. 10.1155/2012/868645 PMC3388584 22778942

[6] Moss SF (2017) The clinical evidence linking helicobacter pylori to gastric cancer. Cell Mol Gastroenterol Hepatol 3, 183–191. 28275685 10.1016/j.jcmgh.2016.12.001 PMC5331857

[7] Bhullar KS , Lagarón NO , McGowan EM , Parmar I , Jha A , Hubbard BP and Rupasinghe HPV (2018) Kinase‐targeted cancer therapies: progress, challenges and future directions. Mol Cancer 17, 48. 29455673 10.1186/s12943-018-0804-2 PMC5817855

[8] Wang L , Yang Q , Peng S and Liu X (2019) The combination of the glycolysis inhibitor 2‐DG and sorafenib can be effective against sorafenib‐tolerant persister cancer cells. Onco Targets Ther 12, 5359–5373. 31371980 10.2147/OTT.S212465 PMC6635829

[9] Wang H , Quan H and Lou L (2017) AKT is critically involved in the antagonism of BRAF inhibitor sorafenib against dabrafenib in colorectal cancer cells harboring both wild‐type and mutant (V600E) BRAF genes. Biochem Biophys Res Commun 489, 14–20. 28536078 10.1016/j.bbrc.2017.05.110

[10] Roviello G , Ravelli A , Polom K , Petrioli R , Marano L , Marrelli D , Roviello F and Generali D (2016) Apatinib: a novel receptor tyrosine kinase inhibitor for the treatment of gastric cancer. Cancer Lett 372, 187–191. 26797419 10.1016/j.canlet.2016.01.014

[11] Benzi G , Camasses A , Atsunori Y , Katou Y , Shirahige K and Piatti S (2020) A common molecular mechanism underlies the role of Mps1 in chromosome biorientation and the spindle assembly checkpoint. EMBO Rep 21, e50257. 32307893 10.15252/embr.202050257 PMC7271318

[12] Silva RD , Mirkovic M , Guilgur LG , Rathore OS , Martinho RG and Oliveira RA (2018) Absence of the spindle assembly checkpoint restores mitotic fidelity upon loss of sister chromatid cohesion. Curr Biol 28, 2837–2844. 30122528 10.1016/j.cub.2018.06.062 PMC6191932

[13] Lim G and Huh WK (2007) Rad52 phosphorylation by Ipl1 and Mps1 contributes to Mps1 kinetochore localization and spindle assembly checkpoint regulation. PNAS 114, E9261–E9270. 10.1073/pnas.1705261114 PMC5676883 29078282

[14] Salvatore G , Nappi TC , Salerno P , Jiang Y , Garbi C , Ugolini C , Miccoli P , Basolo F , Castellone MD , Cirafici AM *et al* (2007) A cell proliferation and chromosomal instability signature in anaplastic thyroid carcinoma. Cancer Res 67, 10148–10158. 17981789 10.1158/0008-5472.CAN-07-1887

[15] Tang J , Lu M , Cui Q , Zhang D , Kong D , Liao X , Ren J , Gong Y and Wu G (2019) Overexpression of ASPM, CDC20, and TTK confer a poorer prognosis in breast cancer identified by gene co‐expression network analysis. Front Oncol 9, 310. 31106147 10.3389/fonc.2019.00310 PMC6492458

[16] Zheng L , Chen Z , Kawakami M , Chen Y , Roszik J , Mustachio LM , Kurie JM , Villalobos P , Lu W , Behrens C *et al* (2019) Tyrosine threonine kinase inhibition eliminates lung cancers by augmenting apoptosis and polyploidy. Mol Cancer Ther 18, 1775–1786. 31358662 10.1158/1535-7163.MCT-18-0864

[17] Liu X , Liao W , Yuan Q , Ou Y and Huang J (2015) TTK activates Akt and promotes proliferation and migration of hepatocellular carcinoma cells. Oncotarget 6, 34309–34320. 26418879 10.18632/oncotarget.5295 PMC4741454

[18] Libouban MAA , de Roos JADM , Uitdehaag JCM , Willemsen‐Seegers N , Mainardi S , Dylus J , Man J , Tops B , Meijerink JPP , Storchová Z *et al* (2017) Stable aneuploid tumors cells are more sensitive to TTK inhibition than chromosomally unstable cell lines. Oncotarget 8, 38309–38325. 28415765 10.18632/oncotarget.16213 PMC5503534

[19] Choi M , Min YH , Pyo J , Lee CW , Jang CY and Kim JE (2017) TC Mps1 12, a novel Mps1 inhibitor, suppresses the growth of hepatocellular carcinoma cells via the accumulation of chromosomal instability. Br J Pharmacol 174, 1810–1825. 28299790 10.1111/bph.13782 PMC5446587

[20] Xie R , Wang P , Cheng M , Sapolsky R , Ji X and Zhao H (2014) The mTOR cell signaling pathway contributes to the protective effects of ischemic postconditioning against stroke. Stroke 45, 2769–2776. 25013017 10.1161/STROKEAHA.114.005406 PMC4146669

[21] Lara‐Gonzalez P , Westhorpe FG and Taylor SS (2012) The spindle assembly checkpoint. Curr Biol 22, R966–R980. 23174302 10.1016/j.cub.2012.10.006

[22] Xie Y , Wang A , Lin J , Wu L , Zhang H , Yang X , Wan X , Miao R , Sang X and Zhao H (2017) Mps1/TTK: a novel target and biomarker for cancer. J Drug Target 25, 112–118. 27819146 10.1080/1061186X.2016.1258568

[23] Yu ZC , Huang YF and Shieh SY (2016) Requirement for human Mps1/TTK in oxidative DNA damage repair and cell survival through MDM2 phosphorylation. Nucleic Acids Res 44, 1133–1150. 26531827 10.1093/nar/gkv1173 PMC4756815

